# Yeast Actin-Related Protein ARP6 Negatively Regulates *Agrobacterium*-Mediated Transformation of Yeast Cell

**DOI:** 10.1155/2015/275092

**Published:** 2015-09-06

**Authors:** Yumei Luo, Zikai Chen, Detu Zhu, Haitao Tu, Shen Quan Pan

**Affiliations:** ^1^Key Laboratory for Major Obstetric Diseases of Guangdong Province, Key Laboratory of Reproduction and Genetics of Guangdong Higher Education Institutes, The Third Affiliated Hospital of Guangzhou Medical University, Guangzhou 510150, China; ^2^Department of Biological Sciences, National University of Singapore, Singapore 117543; ^3^Faculty of Health Sciences, University of Macau, Macau

## Abstract

The yeasts, including *Saccharomyces cerevisiae* and *Pichia pastoris*, are single-cell eukaryotic organisms that can serve as models for human genetic diseases and hosts for large scale production of recombinant proteins in current biopharmaceutical industry. Thus, efficient genetic engineering tools for yeasts are of great research and economic values. *Agrobacterium tumefaciens*-mediated transformation (AMT) can transfer T-DNA into yeast cells as a method for genetic engineering. However, how the T-DNA is transferred into the yeast cells is not well established yet. Here our genetic screening of yeast knockout mutants identified a yeast actin-related protein *ARP6* as a negative regulator of AMT. *ARP6* is a critical member of the SWR1 chromatin remodeling complex (SWR-C); knocking out some other components of the complex also increased the transformation efficiency, suggesting that *ARP6* might regulate AMT via SWR-C. Moreover, knockout of *ARP6* led to disruption of microtubule integrity, higher uptake and degradation of virulence proteins, and increased DNA stability inside the cells, all of which resulted in enhanced transformation efficiency. Our findings have identified molecular and cellular mechanisms regulating AMT and a potential target for enhancing the transformation efficiency in yeast cells.

## 1. Introduction

Yeast is one of the simplest eukaryotic organisms classified in the kingdom Fungi. The budding yeast,* Saccharomyces cerevisiae*, is widely used as human genetic disease models as it is compatible with high throughput screening assays.* Pichia pastoris* is a better host for larger scale recombinant protein production in biopharmaceutical industry than* Escherichia coli* as many human proteins are subject to specific posttranslational modifications in eukaryotic cells. However, all these applications require safe and efficient genetic engineering of yeast cells.

*Agrobacterium tumefaciens* is a soil-borne bacterium, which is frequently utilized as the tool-of-choice for production of genetically modified plants for a very broad range of species [1]. Later study found that* Agrobacterium* is also capable of transforming yeast cells by integrating its T-DNA into the host genome [2]. Generally, the* Agrobacterium*-mediated transformation (AMT) procedures for plants and yeasts are similar, but each organism has its own conditions for the optimal transformation efficiency. Thus, a better understanding of the mechanism of AMT in yeast cells will lead to further refinement of this system for yeast engineering.

*Agrobacterium* can transfer not only DNA but also proteins into plant and yeast cells via the well-conserved bacterial type IV secretion system [3].* Agrobacterium* virulence protein VirD2 is an endonuclease that directly processes the single-stranded T-DNA to be delivered into recipient cells. VirD2 also covalently binds to the 5′ end of the T-DNA and guides its transfer from the bacteria to the recipient cell [4]. Another virulence protein VirE2 binds to the T-DNA along the entire sequence, which leads to formation of the T-complex [5]. T-complex is delivered into recipient cells by the VirB/VirD4 machinery. Following the entry into the host cytoplasm, the T-complex is most likely transported through the cytoplasm to the nucleus in the form of VirD2-T-DNA coated by VirE2. The cytoplasmic transport of T-complex is one of the most obscured parts in the AMT process. Because of the very large size of the T-complex and the dense structure of the host cytoplasm which may block simple diffusion of macromolecules, it was hypothesized that T-complex transport was an active process involving the host microtubule network [6]. In 2005, Salman et al. utilized a single-particle tracking method to show that an artificial VirE2-T-DNA complex moved along microtubules* in vitro*, which required nuclear localization signal peptides and was blocked by inhibition of the minus-end directed dynein [7]. Nevertheless, more evidence is required to support the assumption that microtubule is important for the AMT process.

Here our genetic screening of yeast knockout genes associated with microtubules identified* ARP6* as a negative regulator of the AMT process.* ARP6* is a crucial component of the SWR1 chromatin remodeling complex (SWR-C), which exchanges the conventional histone H2A to the histone variant H2A.Z (*Htz1* in yeast) [8]. Previous studies have shown that some histones and chromatin remodeling complexes [9] are crucial for the AMT process. For example, H2A is essential for T-DNA integration in somatic cells [10] and is highly expressed in the tissues susceptible to* Agrobacterium* infection [11]. H2A may contribute to a more relaxed structure of the host genome, thus facilitating the inserting of T-DNA.

The gene functions of* ARP6* which significantly affects transformation efficiency were extensively studied with a combination of genetic, biochemical, and bioimaging approaches in this study. We examined the roles of* ARP6* in microtubule integrity, virulence protein transport and degradation, and T-DNA trafficking in order to provide more insight into the molecular mechanism of the AMT process. All these results indicate that* ARP6* is a negative regulator with multiple effects on* Agrobacterium*-mediated genetic transfer.

## 2. Materials and Methods

### 2.1. Plasmids, Strains, and Fluorescent Microscopy

The plasmids used in this study are listed in Table S1 (see Supplementary Material available online at http://dx.doi.org/10.1155/2015/275092).* A. tumefaciens* EHA105 (pHT101) were grown at 28°C in MG/L medium, induced in IBPO_4_ with kanamycin and acetosyringone [12].* S. cerevisiae* BY4741 (*MATa*,* his3Δ1*,* leu2Δ0*,* met15Δ0*, and* ura3Δ0*) were grown at 28°C in YPD, while transformed yeast cells were grown on SD medium with appropriate amino acid dropout.

Olympus Fluoview FV1000 was used to detect signals emitted by fluorescent dyes or proteins. The excitation lights for DAPI signal, Cy3 signal, and signal of green fluorescent protein (GFP) were 405 nm, 543 nm, and 488 nm, respectively. The images were captured and analyzed with Olympus Fluoview vir1.6b.

### 2.2. Split-GFP Assay

The split-GFP assay in this study was carried out as previously described [13]. In brief, GFP was divided into a small fragment (S11) and a large fragment (S1–10). The two fragments emit fluorescence when combined but either one independently cannot. The gene to the small fragment was fused with* virE2* gene in a permissive site so that it would not affect the translocation of VirE2 from* Agrobacterium* to yeast cells. The large one was expressed in the yeast cells before AMT. When the AMT takes place, during which VirE2 enters the yeast cells, the two fragments form a functional GFP and emit green signals. Then, the amount of VirE2 aggregates could be quantified by counting the green dots using fluorescent microscope.

### 2.3. FISH Assay

The FISH in this study was carried out as previously described [14] with some modifications, including the design of probes (Figure S2), RNase treatment, and denaturing process. Before hybridization, the samples were incubated with 2× SSC containing 0.1 mg/mL RNase at 37°C for 1 h and with 40% formamide/2× SSC at 80°C for 30 min. These two steps were to fully remove RNA molecules and denaturation of the T-DNA to facilitate hybridization.

### 2.4. DNase Activity Assay

Before the preparation of cell lysates, 2 × 10^6^ yeast cells for each droplet were resuspended in 100 *μ*L IBPO_4_ and dropped onto CM plates and incubated at 20°C for 24 h. This was to mimic the cocultivation conditions so that the results could reflect the DNase activity during AMT process. The extraction of cell lysates was performed as described previously [15], with some modification in the lysis buffer (150 mM NaCl, 50 mM Tris-HCl, pH 7.4, 1 mM PMSF, and 1 : 1000 protease inhibitor cocktail (Sigma P8340)). Cells were washed off from the CM plates and the OD_600_ was measured. 4 OD of cells were collected and resuspended in 100 *μ*L lysis buffer which contains 0.1 g glass beads. The mixture was vigorously vortexed for 4 min to produce the cell lysates and then spun down and kept on ice. 10 *μ*L, 20 *μ*L, and 30 *μ*L cell lysates were added with 1 *μ*g plasmid DNA (pHT105-ARP6) and 2 mM MgCl_2_, supplemented with lysis buffer to total volume of 40 *μ*L, and incubated at 37°C for 1 h. DNA fragments were separated on 1% agarose gel.

### 2.5. Immunofluorescence Assay

The yeast cells were cultured in YPD medium at 28°C and fixed with 4% paraformaldehyde at room temperature for 1 h. The cells were treated with 0.5 U/*μ*L lyticase at 30°C for 30 min, attached to polylysine coated cover-slips, and stored in 70% ethanol at −20°C. After rehydration with PBS, the samples were incubated with blocking solution (0.1% triton 100, 1% BSA, and PBS) at room temperature for 1 h. 1 : 25 rat anti-*α*-tubulin antibody (AbD Serotec) in blocking solution was added and incubated for 1 h. Washing was conducted using PBS for 5 min 3 times before incubating with 1 : 200 goat anti-rat IgG DyLight 488 (AbD Serotec) for 1 h. The samples were then washed again with PBS 3 times and stained with DAPI.

## 3. Results

### 3.1. Knockout of* ARP6* Consistently and Significantly Increases AMT Efficiency

**Figure 1:**
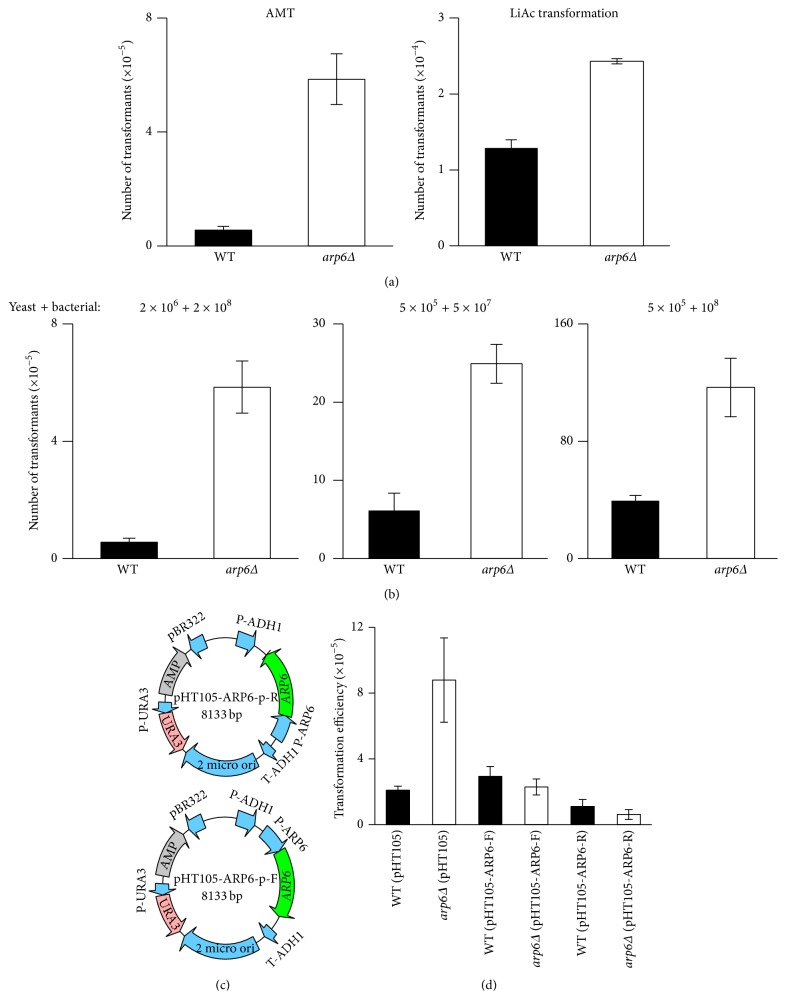
Knockout of ARP6 increases AMT efficiency. (a) Transformation efficiency comparison between AMT and LiAc transformation. (b) The transformation efficiencies of WT and* arp6Δ* under different conditions. The* arp6Δ* mutant consistently increased transformation efficiencies as compared to WT with different input numbers and ratios of yeast to* Agrobacterium*. (c) The plasmids containing* ARP6* that were introduced into yeast before AMT. (d) Complementation and overexpression assays. All the results were averaged from three independent experiments. Error bars present for SD. ^*∗*^ *P* < 0.05 by *t*-test.

We determined the effect of* ARP6* on AMT efficiency by performing cocultivation of yeast and* Agrobacterium* according to the standard protocol [2] with minor modification. To test the specificity of this effect, AMT was compared with LiAc transformation. It is clearly shown that the effect of* ARP6* was much more significant in AMT process than LiAc transformation, as the elevated fold change for AMT was above 10 times while being only 2 times for LiAc transformation (Figure 1(a)). These results suggest that the knockout effect of* ARP6* is more competent to AMT.

Because the number of transformants is dramatically affected by the input numbers and ratios of yeast cells to* Agrobacterium*, three conditions (number of* Agrobacterium* + number of yeasts) were adopted to confirm the effect of* ARP6*: (1) 2 × 10^8^ + 2 × 10^6^ (100 : 1); (2) 5 × 10^7^ + 5 × 10^5^ (100 : 1); (3) 1 × 10^8^ + 5 × 10^5^ (200 : 1). The results showed that the number of transformants of* arp6Δ* is consistently and significantly higher than that of the WT strain, indicating that* ARP6* is indeed involved in negatively regulating the AMT process (Figure 1(b)).

In order to further confirm the effect of* ARP6* in AMT process, the complementation and overexpression assays were performed.* ARP6* with its own promoter was inserted into the binary vector pHT105 in both directions (Figure 1(c)). The constructs were introduced by LiAc transformation into the* arp6Δ* mutant and WT strain, respectively. The transformed yeasts were then tested for AMT efficiency. It can be seen that the empty vector did not influence the effect of* ARP6* knockout; the mutant still has significantly increased transformation efficiency as compared to WT. On the other hand, both constructs successfully complemented the loss of* ARP6* in the mutant, since the transformation efficiency is similar to or even lower than that of the WT strain. For the overexpression assay, the forward construct did not have much effect probably due to lower expression of* ARP6*, while the reverse reduced the transformation efficiency by 2-fold. Although the forward construct did not affect AMT in WT, it indeed successfully complemented the mutant. The complementation and overexpression assays further confirm the role of* ARP6* in negatively regulating the AMT process (Figure 1(d)).

### 3.2. *ARP6* Regulates AMT Process through SWR-C

Since ARP6 is a key component of the SWR1 chromatin remodeling complex (SWR-C) that exchanges histone H2A with H2A.Z (*Htz1* in yeast) [8], the increased transformation may be due to the loss-of-function of this complex. Previous studies have shown that H2A is required for the* Agrobacterium*-mediated tumorigenesis and the stable genetic transformation of* Arabidopsis* [10, 11]. Considering the fact that the structures and functions of H2A and the SWR-C are highly conserved in eukaryotic organisms [16], it is reasonable to hypothesize that the SWR-C in yeast also functions to counteract AMT.

**Figure 2:**
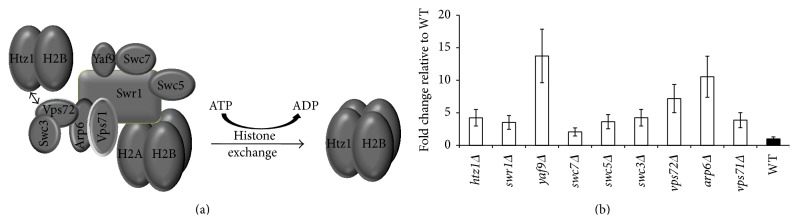
Changes of AMT efficiency of SWR-C mutants and* htz1Δ* as compared to WT. (a) Structure of SWR-C. (b) The input numbers of* Agrobacterium* and yeast are 2 × 10^8^ and 2 × 10^6^, respectively. The results were averaged from three independent experiments. Error bars present for SD. ^*∗*^ *P* < 0.05 by *t*-test.

Within the SWR-C, there are 14 components, 6 of which are required for the survival of yeast and cannot be knockout (Figure 2(a)). In order to find out whether the SWR-C was involved in the T-DNA transfer process, AMT efficiencies of the other 8 SWR-C knockout mutants and the* htz1Δ* were tested and the fold changes of transformation efficiency as compared to WT were shown in Figure 2(b). The results showed that the efficiencies for all the mutants were increased from 2 to 10 times as compared to WT, indicating that this chromatin remodeling complex indeed plays an important role in reducing the success rate of AMT. More interestingly, it is noticeable that the increased transformation efficiency for* arp6Δ* is much higher than those of* htz1Δ* and* swr1Δ* mutants, indicating that* ARP6* may further affect the AMT process via other pathways besides histone exchange.

### 3.3. Loss of* ARP6* Results in Higher Import Rate of VirE2

**Figure 3:**
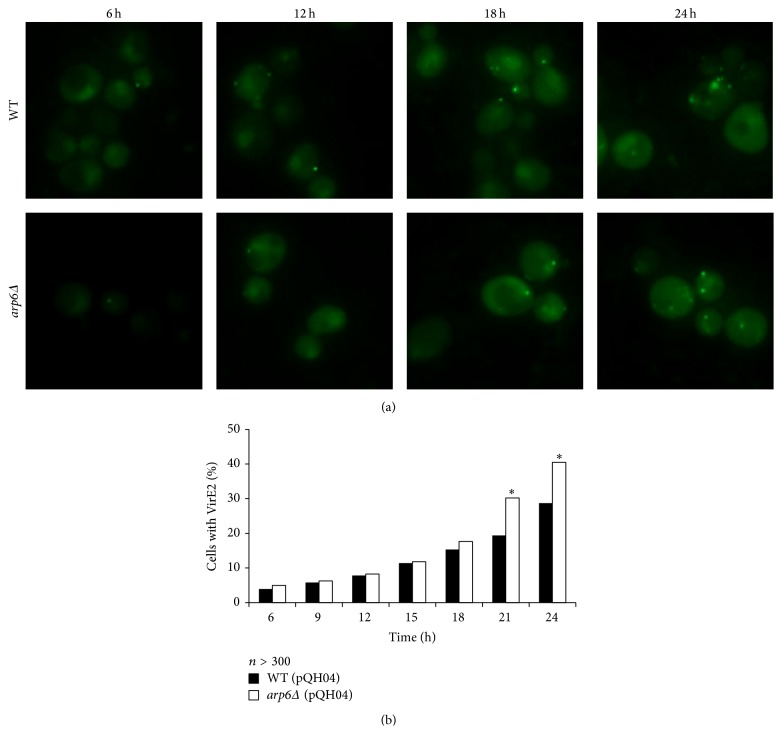
Split-GFP assay to monitor VirE2 import into yeast cells during AMT process. (a) The VirE2 transport rate and the number of VirE2 aggregates in each cell increase during the 24-hour time course in both WT and* arp6Δ* mutant. (b) The import rates were similar for both strains at the beginning, but the percentage was much higher in the mutant after 21 h. The vector pQH04 in the yeast cells contains the large fragment of GFP. Error bars present for SD. ^*∗*^ *P* < 0.05 by *t*-test.

In order to find out whether the higher transformation efficiency for* arp6Δ* is due to more virulence proteins imported into the yeast cells, the split-GFP assay for VirE2 was performed. To better monitor the transport of VirE2, 3-hour interval time course of AMT was carried out. The results of VirE2 tracking were shown in Figure 3(a) and the import rates in WT and* arp6Δ* were calculated by counting more than 300 yeast cells for each time point (Figure 3(b)).

As can be seen, the VirE2 aggregates emitting green fluorescence could be detected with fluorescence microscopy in the yeast cells. The green dots within each cell grew in number as time went on (Figure 3(a)); in addition, the percentage of cells harboring VirE2 increased as well for both WT and* arp6Δ* (Figure 3(b)). This result indicates that the transport of virulence proteins from* Agrobacterium* to the yeast cells starts at the very early stage of cocultivation and is a continuous process. Figure 3(b) shows the percentage of cells containing VirE2 aggregates for both strains during the time course. It can be seen that the import rates of virulence proteins were similar at the beginning of cocultivation (before 12 h); however, the VirE2 import rate grew faster for* arp6Δ* (pQH04) at the later stage. After 24-hour cocultivation, there were around 41% of cells carrying VirE2 for* arp6Δ* (pQH04), while being only 29% for WT (pQH04). These data show that APR6 knockout tended to uptake VirE2 more easily at the later stage of the AMT process, suggesting that virulence proteins import could be inhibited by the gene functions of* ARP6*.

### 3.4. *ARP6* Regulates VirD2 Degradation in the Yeast Nucleus

**Figure 4:**
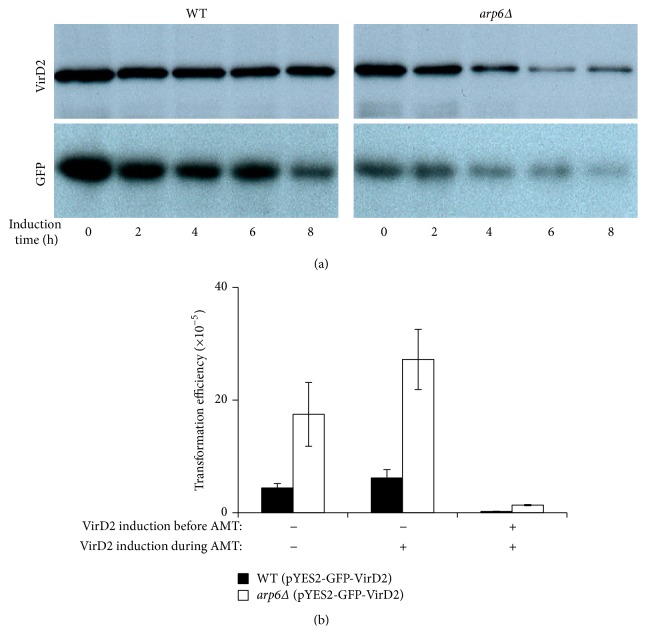
Effects of VirD2 degradation in WT and* arp6Δ* on AMT efficiency. (a) Time course experiment for VirD2-GFP fusion protein and GFP degradation in WT and arp6Δ after inhibition of new protein synthesis. The yeast cells were cultivated in SD ura-galactose medium overnight before addition of cycloheximide to the final concentration of 10 *μ*g/mL. (b) The effect of VirD2 induction on AMT efficiency. The results were averaged from three independent experiments. Error bars present for SD. ^*∗*^ *P* < 0.05 by *t*-test.

We then looked into the effect of* ARP6* on virulence protein degradation. Protein degradation occurs followed by the cytoplasmic transport and nuclear targeting of the T-complex. Virulence proteins degradation after nuclear import was crucial for T-DNA processing and integration [17]; thus, reduction of protein degradation may attenuate transformation efficiency. In order to see whether* ARP6* will affect virulence protein degradation, VirD2 was overexpressed in galactose medium in both WT and* arp6Δ* mutant before adding cycloheximide to stop new protein synthesis. The samples were taken every 2 h and the result of Western Blot showed that VirD2 degradation is enhanced with the knockout of* ARP6* during the time course (Figure 4(a)).

In order to see whether VirD2 inside the host cell could truly affect the genetic transfer by* Agrobacterium*, the transformation efficiency of WT (pYES2-GFP-VirD2) and* arp6Δ* (pYES2-GFP-VirD2) was tested with or without expression of VirD2. pYES2 is an inducible vector with a Gal1 promoter so that VirD2 expression could be controlled by change of medium from glucose to galactose. It can be seen that VirD2 expression during cocultivation slightly increased the transformation efficiency. However, VirD2 expression before cocultivation dramatically decreased efficiency in both WT and* arp6Δ* (Figure 4(b)). This intriguing phenomenon suggests that, during cocultivation, production of VirD2 may facilitate nucleus-targeting of the T-complex, while overexpression of VirD2 prior to cocultivation affects virulence protein degradation, thus reducing transformation efficiency.

### 3.5. The Disrupted Microtubule Structure in* arp6Δ* Is Beneficial for AMT

After the import of T-complex, the cytoplasmic transport of virulence factors in the host cells is required for successful transformation. Many pathogens that cause widespread illness depend on microtubules for efficient nuclear targeting and successful infection, such as human immunodeficiency virus [18], human cytomegalovirus [19], and human papillomavirus [20]. An* in vitro* study also suggested the involvement of microtubules and related motors in transport of T-complex [7].

**Figure 5:**
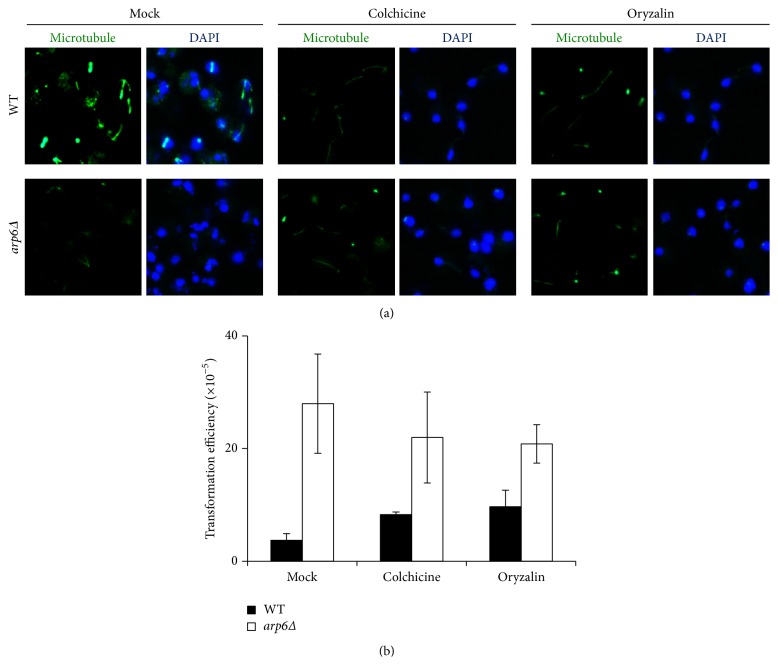
The disrupted microtubule network in* arp6Δ* was beneficial for AMT process. (a) The loss of* ARP6* resulted in disrupted microtubule structure which is similar to the effect of colchicine/oryzalin treatment. (b) The effect of colchicine and oryzalin on AMT efficiency. 5 × 10^5^ yeast cells and 10^8^ bacteria were cocultivated at 20°C for 24 h. The results were the average of three independent experiments. Error bars present for SD. ^*∗*^ *P* < 0.05 by *t*-test.

In order to see whether* ARP6* affects the intracellular transport of virulence factors during AMT process, immunofluorescence was used to detect microtubule structures of WT and* arp6Δ* mutant. As can be seen in Figure 5(a), the microtubule network is intact in WT strain. Without* ARP6*, the microtubules were disrupted, becoming fewer and shorter. On the other hand, when the yeast cells were treated with microtubule-depolymerization drugs (colchicine or oryzalin), the microtubule networks in both WT and* arp6Δ* yeasts showed similar disrupted phenotypes (Figure 5(a)).

The microtubule-depolymerization drugs had different effect on the AMT efficiency for WT and* arp6Δ*. For WT which has intact microtubule structures and lower transformation efficiency, the addition of colchicine or oryzalin enhanced the AMT efficiency by 2- and 3-fold. For* arp6Δ* which contains disrupted microtubule structures, the addition of colchicine or oryzalin did not have much effect on the AMT efficiency (Figure 5(b)). These results indicate that the complex structure of microtubules is an inhibitory factor for the cytoplasmic transport of T-complex and further confirm that the disrupted microtubules in* arp6Δ* mutant are beneficial for the AMT process.

### 3.6. *ARP6* Affects Stability but Not the Uptake of T-DNA by Regulation of Nuclease Activity

Next, we investigate whether ARP6 affects the delivery of T-DNA in yeast cells. To elucidate the function of* ARP6* in T-DNA trafficking, fluorescent* in situ* hybridization (FISH) assay was performed. Four DNA probes complementary to the GFP fragment of T-DNA were designed to perform the FISH assay. Four thymines (T) in each probe were replaced with Cy3 dyes so that labeled DNA could be detected by fluorescence microscopy (Figure S1). The probes can specifically bind to single-stranded DNA but not to double-stranded DNA (Figure S2).

**Figure 6:**
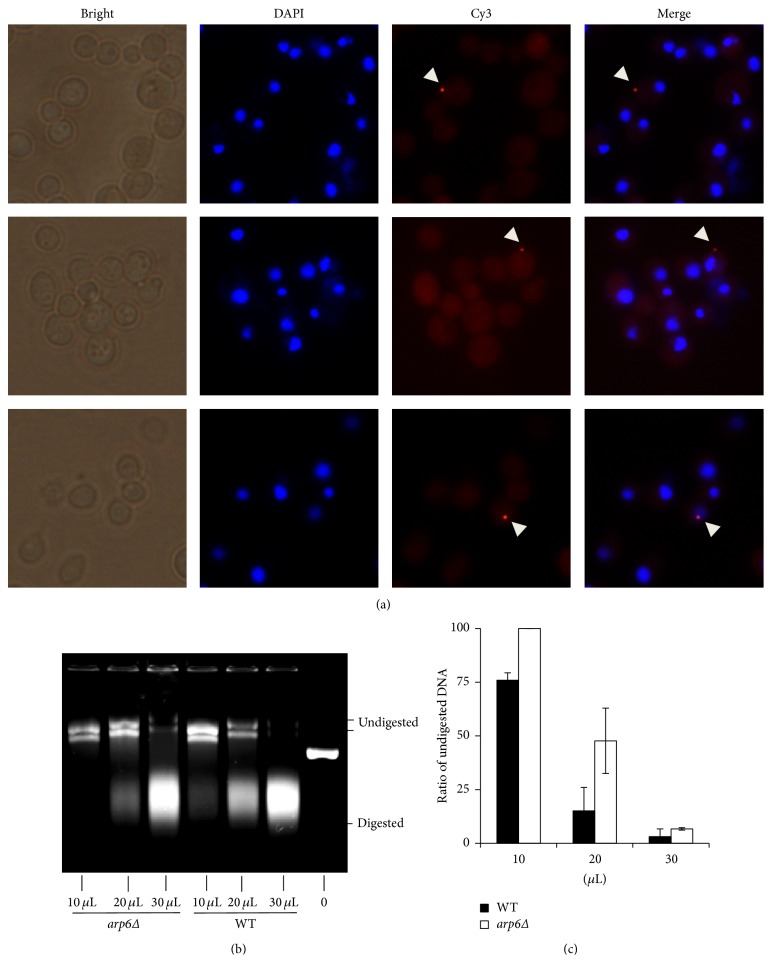
*ARP6* affects stability but not the uptake of T-DNA. (a) FISH results for cocultivation mixture of yeast and* Agrobacterium* for 24 h. T-DNA at the periphery of yeast cell and in the nucleus of the yeast cell was detected by Cy3-labeling probes. DAPI staining indicates the location of yeast nuclei. The arrows indicate the probes hybridized to T-DNA. (b) Nuclease activity assay for WT and* arp6Δ* lysates. Lanes 1–3:* arp6Δ* lysates 10, 20, and 30 *μ*L, respectively; lanes 4–6: WT lysates 10, 20, and 30 *μ*L, respectively; lane 7: undigested plasmid DNA. (c) Normalized data for the activity of DNase analyzed by Image J. Error bars present for SD. ^*∗*^ *P* < 0.05 by *t*-test.

The results of FISH for 24 h cocultivation mixtures (5 × 10^5^ yeasts with 10^8^ bacteria) were shown in Figure 6(a). Most of the signals detected were at the cell periphery (Figure 6(a), top, middle panels) while a small amount was detected within the host nucleus (Figure 6(a), bottom panel). The signal was rarely detected in the cytoplasm. We hypothesize that T-DNA may move towards the nucleus in a high speed that T-DNA could not be detected in the cytoplasm by FISH.

Because of the low transformation efficiency, the detection rate of T-DNA may not be statistically significant (Table S2); but we can still see the general trend of T-DNA uptake. We found that there was not much difference of the uptake rates between WT (0.11%) and* arp6Δ* (0.15%) in about 3000 cells after 24-hour cocultivation. More importantly, the detection rate of T-DNA for the mutant was similar to the transformation efficiency (0.12%) while the detection rate for the WT was much higher than the AMT efficiency (0.04%). These data, taken together, suggest that* ARP6* may not affect the uptake of T-DNA but affects the stability of T-DNA in the host cell.

The percentage of WT cells with T-DNA was higher than the AMT efficiency, which implies that most of T-DNA inside the host cell may be degraded. If so, the DNase activity of the yeast cells may be involved in resistance of the genetic transfer process. The DNase activity assay was carried out as described previously [21]. The DNase activity was enhanced as the amount of cell lysates was added for both strains (Figure 6(b)). In addition, the percentage of digested DNA for the WT was consistently higher as compared to the mutant (Figure 6(c)). This result suggests that the knockout of* ARP6* downregulates the activity of DNase in yeast, which may function as an important part of the defensive pathway for the host cell to destroy foreign DNA. Thus, the higher transformation efficiency for the* arp6Δ* could be partly attributed to the lower DNase activity.

## 4. Discussion

In this study, we have investigated the molecular and cellular mechanisms for* Agrobacterium*-mediated transformation of yeast cells involving the actin-related protein* ARP6*.* ARP6* is commonly found in various kinds of chromatin remodeling and modifying complexes [22], regulating sets of gene activation or silencing. We have shown that* ARP6* is a negative regulator for the AMT process as knockout of* ARP6* consistently and significantly increased transformation efficiency. We further demonstrated that* ARP6* functions partially dependent on SWR-C which exchanges conventional histone H2A with HTZ1.* ARP6* may also act independently of the SWR-C since the knockout effect of* ARP6* was much more significant than those of SWR1 and HTZ1. In a recent study,* ARP6* was found to contribute to chromatin organization and control ribosomal protein gene expression level independently of SWR-C [23].

*ARP6* has been found to be genetically associated with TUB3 that encodes *α*-tubulin, a subunit of microtubules, according to several high-throughput studies [24–26]. The fungal microtubules play important roles in a multitude of classic cellular processes, including cell movement, cell polarity regulation, mitosis, and intracellular organelle transport [27–30]. More interestingly, mRNAs transport was also found to be related to molecular motors and microtubule cytoskeleton [31, 32]. Thus, we examined the effect of* ARP6* on microtubule structure and the relationship between microtubule integrity and AMT efficiency. Microtubules were proposed to be responsible for T-DNA trafficking; however, our study found that the integrity of microtubule structure was a restricted factor for the AMT process and that* ARP6* was required for the integrity. This finding suggests that the dense structure of cytoskeleton may inhibit the movement of T-complex, thus reducing the transformation efficiency.

We also found that two of the virulence proteins transported from* Agrobacterium* were also influenced by the functions of* ARP6*. The split-GFP system for the VirE2 protein import showed that* ARP6* may inhibit the T-DNA transfer by suppressing the transportation of virulence proteins to some extent. Virulence proteins are important for T-DNA transport at the early stage of transformation; on the other hand, virulence proteins degradation after nuclear import is also crucial for T-DNA processing and integration. For example, in plant cell VirF targets VirE2 and the host VIP protein and modulates the ubiquitin-mediated proteasome-dependent degradation [17]. Such degradation could be important for* Agrobacterium* infection since the deletion of VirF or mutations in the F-box motif of VirF substantially decrease the bacterial virulence [33]. VirE2 does not enter the nucleus in yeast cell; thus, we checked the degradation of VirD2 by the VirD2-GFP degradation assays. We revealed that overexpression of VirD2 indeed dramatically reduced transformation efficiency and that ARP6 enhanced the stability of VirD2, which may be a key reason for the low transformation efficiency of WT strain.

One of the most obscure processes in the* Agrobacterium*-mediated gene transfer is the trafficking of the T-DNA inside the host cell. As far as we know, there is no report for the* in vivo* detection of T-DNA in yeast cell, probably due to the low efficiency of transformation. T-DNA tracking is an important yet challenging task for better understanding of the cellular and molecular mechanisms of the AMT process. In this study, we managed to detect and semiquantify T-DNA during the genetic transfer from* Agrobacterium* to yeast cell by FISH. With proper optimization, this technique could also be applied to monitor T-DNA trafficking in plant transformation. More importantly, if the small probes could specifically bind to the single-stranded T-DNA within* Agrobacterium* and be transferred to the host cell, T-DNA delivery can be monitored in a real-time format in the future.

In addition, as* Agrobacterium* can infect human cells but not proliferate in them [34], AMT also holds the potential to be developed as a genetic engineering tool for human cells. A good example is the baculovirus, which is originally an insect virus but is later found to infect human cells as well. Taking advantages of its nonreplicative and nonintegral natures in human cells, baculovirus is often used to genetically modify human stem cells for therapies now [35–40].

## Supplementary Material

Supplementary Figures and Tables: Table S1. Plasmids used in this study; Table S2. The T-DNA detection rates for co-cultivation mixture; Figure S1. The GFP DNA sequence of T-DNA and the 4 probes targeting sites within the GFP sequence; Figure S2. The test for specificity of GFP probes; Figure S3. Cell growth was not affected by the expression of VirD2 protein.
