# Supplementary material for: Yeast Actin-Related Protein ARP6 Negatively Regulates Agrobacterium-Mediated Transformation of Yeast Cell
Source: Biomed Res Int. 2015 Sep 6;2015:275092. doi: 10.1155/2015/275092 (PMC4575723; doi:10.1155/2015/275092)
Supplement: Supplementary file 1 — Supplementary Figures and Tables: Table S1. Plasmids used in this study; Table S2. The T-DNA detection rates for co-cultivation mixture; Figure S1. The GFP DNA sequence of T-DNA and the 4 probes targeting sites within the GFP sequence; Figure S2. The test for specificity of GFP probes; Figure S3. Cell growth was not affected by the expression of VirD2 protein. [file 275092.f1.pdf]

Table S1. Plasmids used in this study

Plasmid	Characteristic	Source or reference
pHT101	Constructed by ligation of vector pCB301and pACT2, in which the *GAL4AD* gene is replaced by EGFP reporter gene; Km^R, Amp^R, 2μ origin, *LEU2*.	Lab collection
pYES2	Yeast expression vector; 2 μ replicon; *GAL1* promoter, *CYC1* terminator; *URA3*, Amp^R	Invitrogen
pHT105	Yeast expression vector; 2 μ replicon; *ADH1* promoter, *ADH1* terminator; *URA3*, Amp^R	This study
pHT105-ARP6pr-F	*ARP6* gene with its natural promoter inserted into pHT105 forwardly	This study
pHT105-ARP6pr-R	*ARP6* gene with its natural promoter inserted into pHT105 reversely	This study
pYES2-GFP	pYES2 carrying a *GFP* reporter gene	Lab Collection
pYES2-GFP-VirD2	pYES2 carrying a GFP-N terminal VirD2 fusion sequence	Lab Collection

Table S2. The T-DNA detection rates for co-cultivation mixture.

Co-cultivation mixture	No. of Cy3 signals	No. of cells counted	Percentage($\times 10^{-4}$)	Transformation efficiency ($\times 10^{-4}$)
Agrobacterium-WT	3	2815	10.7	3.9
Agrobacterium -arp6Δ	5	3383	14.8	11.7

Agrobacterium -arp6Δ	No. of Cy3 signals	No. of cells counted	Percentage($\times 10^{-4}$)
8 h	3	3096	9.7
24 h	7	4701	14.9
48 h	4	2310	17.3

```
1     TTATTTGTAC AATTCATCCA TACCATGGGT AATACCAGCA GCAGTAACAA ATTCTAACAA
61    GACCATGTGG TCTCTCTTTT CGTTTGGATC TTTGGATAAG GCAGATTGAG TGGATAAGTA
121   ATGGTTGTCT GGTAACAAGA CTGGACCATC ACCAATTGGA GTATTTTGTT GATAATGGTC
181   AGCTAATTGA ACAGAACCAT CTTCAATGTT GTGTCTAATT TTGAAGTTAA CTTTGATACC
241   ATTCTTTTGT TTGTCAGCCA TGATGTAAAC ATTGTGAGAG TTATAGTTGT ATTCCAATTT
301   GTGACCTAAA ATGTTACCAT CTTCTTTAAA ATCAATACCT TTTAATTCGA TTCTATTAAC
361   TAAGGTATCA CCTTCAAACT TGACTTCAGC TCTGGTCTTG TAGTTACCGT CATCTTTGAA
421   AAAAATAGTT CTTTCTTGAA CATAACCTTC TGGCATGGCA GACTTGAAAA AGTCATGTTG ← Probe 2
481   TTTCATATGA TCTGGGTATC TAGAAAAACA TTGAACACCA TAAGTTAAAG TAGTGACTAA ← Probe 3
541   GGTTGGCCAT GGAACTGGCA ATTTACCAGT AGTACAAATA AATTTTAAGG TCAATTTACC ← Probe 4
601   GTAAGTAGCA TCACCTTCAC CTTCACCGGA GACAGAAAAT TTGTGACCAT TAACATCACC ← Probe 5
661   ATCTAATTCA ACCAAAATTG GGACAACACC AGTGAATAAT TCTTCACCTT TAGACAT
```

Figure S1. The GFP DNA sequence of T-DNA and the 4 probes targeting sites within the GFP sequence.

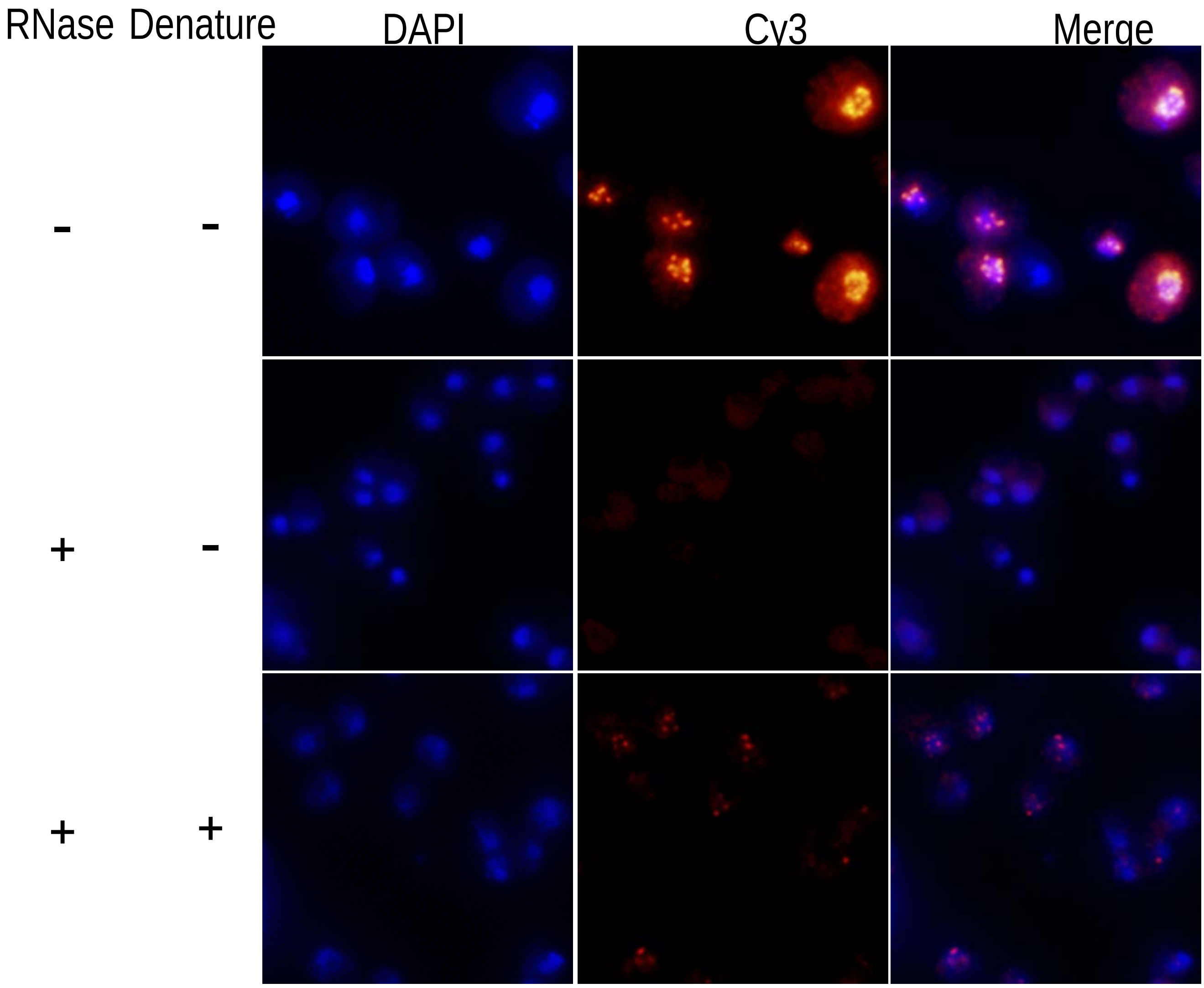

Figure S2. The test for specificity of GFP probes. RNAs within the yeast cells could be detected by the probes without denature and RNase treatment. No signals could be detected with RNase treatment. However, the signals could be detected again after treatment of RNase and denature, which means the probes can specifically bind to denatured DNA.

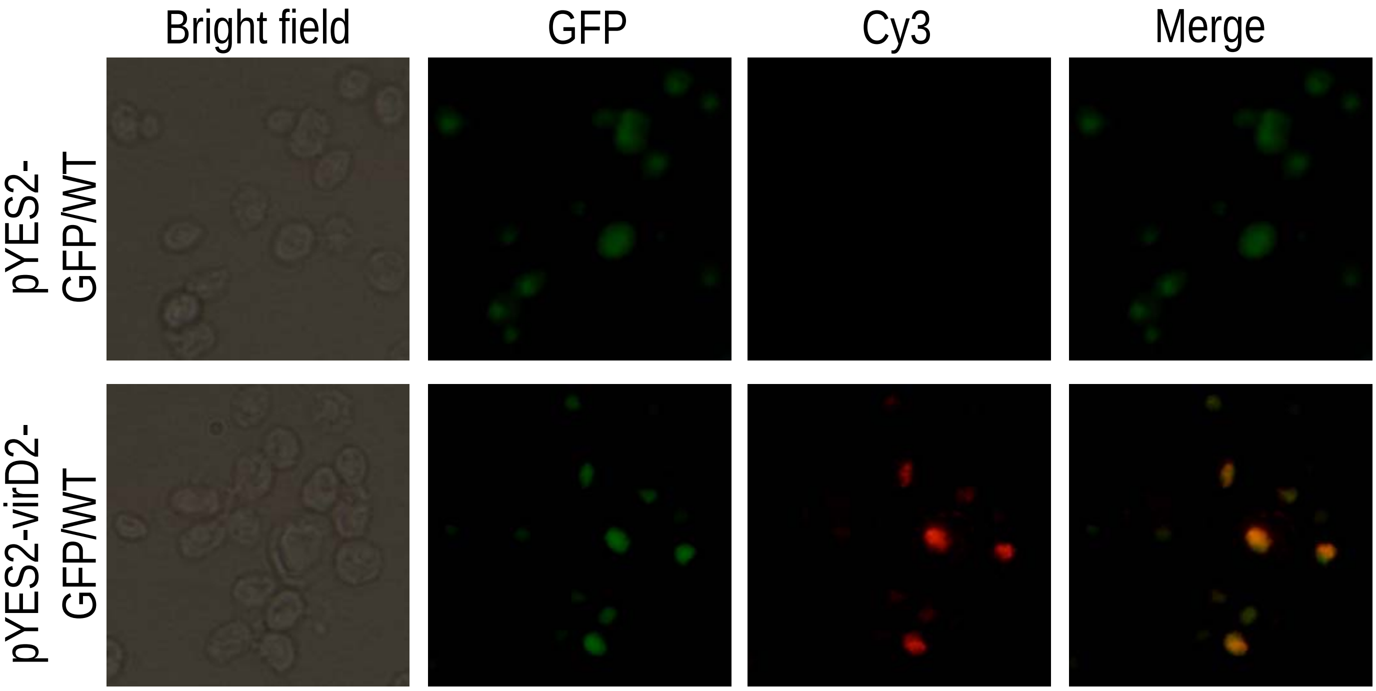

Figure S3. Cell growth was not affected by the expression of VirD2 protein.
